# Insane in the vembrane: filtering and transforming VCF/BCF files

**DOI:** 10.1093/bioinformatics/btac810

**Published:** 2022-12-15

**Authors:** Till Hartmann, Christopher Schröder, Elias Kuthe, David Lähnemann, Johannes Köster

**Affiliations:** Algorithms for Reproducible Bioinformatics, University Hospital Essen, University of Duisburg-Essen, Essen 45147, Germany; Genome Informatics, Institute of Human Genetics, University Hospital Essen, University of Duisburg-Essen, Essen 45147, Germany; Computer Science XI, TU Dortmund University, Dortmund 44227, Germany; Algorithms for Reproducible Bioinformatics, University Hospital Essen, University of Duisburg-Essen, Essen 45147, Germany; Department of Medical Oncology, West German Cancer Center, University Hospital Essen, University of Duisburg-Essen, Essen 45147, Germany; Algorithms for Reproducible Bioinformatics, University Hospital Essen, University of Duisburg-Essen, Essen 45147, Germany; Department of Medical Oncology, Dana-Farber Cancer Institute, Harvard Medical School, Boston, MA 02215, USA

## Abstract

**Summary:**

We present vembrane as a command line variant call format (VCF)/binary call format (BCF) filtering tool that consolidates and extends the filtering functionality of previous software to meet any imaginable filtering use case. Vembrane exposes the VCF/BCF file type specification and its inofficial extensions by the annotation tools *VEP* and *SnpEff* as Python data structures. vembrane filter enables filtration by Python expressions, requiring only basic knowledge of the Python programming language. vembrane table allows users to generate tables from subsets of annotations or functions thereof. Finally, it is fast, by using pysam and relying on lazy evaluation.

**Availability and implementation:**

Source code and installation instructions are available at github.com/vembrane/vembrane (doi: 10.5281/zenodo.7003981).

**Supplementary information:**

Supplementary data are available at *Bioinformatics* online.

## 1 Introduction

Identifying variation from DNA- or RNA-sequencing data and determining its effect on phenotypes are at the heart of a wide range of biological and medical research efforts. Initial bioinformatics processing of such sequencing data obtains thousands to millions of individual differences between one or more biological samples and their reference genome. These variants are annotated with data properties and known or predicted phenotypic effects and usually stored in the variant call format (VCF) or its binary equivalent (BCF) (Danecek *et al.*, 2011). This annotation information can then be used to filter down to a set of interesting candidate variants, for example, those known to be drug targets in a specific disease.

**Fig. 1.**
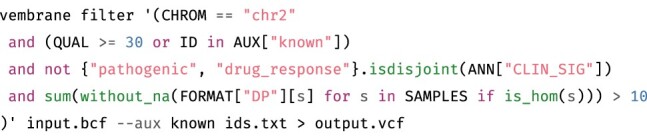
Example invocation of vembrane filter. The file ids.txt contains one ID per line and is parsed as a set. In plain English, the filter expression translates to ‘keep all records from chromosome 2 where the quality is at least 30 or the ID is in the set of known IDs, and where at least “pathogenic” or “drug_response” is part of the clinical significance annotations, and where the sum of read depths across all samples that report a homozygous genotype is at least 10’

Here, we present *vembrane*, a new filtering tool for all versions of the VCF and BCF formats. *vembrane* consolidates and extends the functionality of previously available tools and uses standard Python syntax, while achieving very good processing speed. The direct use of Python syntax enables flexible and powerful expressions (Fig. 1) and obviates the need to adapt to a new syntax for users already familiar with Python. It supports both *SnpEff* (Cingolani *et al.*, 2012; Ruden *et al.*, 2012) and *VEP* (McLaren *et al.*, 2016) annotations out of the box and has an extensible design which allows easy integration of new annotation sources. To our knowledge, it is the only variant filtering tool that can handle groups of breakend events that represent structural variants. It consists of three subcommands for processing VCF records: filter for filtering, table for converting into a tabular format and annotate for adding additional annotations based on genomic ranges.

## 2 Materials and methods

### 2.1 Implementation

For each record, *vembrane* evaluates a given Python expression which has access to all fields defined in the VCF specification as local variables (CHROM, POS, ID, REF, ALT, QUAL, FILTER, INFO, FORMAT), typed according to the VCF file’s header and parsed via pysam. Variant annotation is available via the ANN dictionary. Annotations from *SnpEff* and *VEP* have custom parsers in vembrane, making them easy and safe to use in filter expressions. For VCF records with multiple ANN annotation fields (for multiple affected transcripts), vembrane only keeps ANN entries that match the expression. The respective record is only kept if at least one ANN passes the filter expression [an implicit any, i.e. conceptually: any(evaluate(expr, ann) for ann in annotations), see Supplementary Fig. S2].

Records with multiple alternative alleles may have completely unrelated annotations. This both complicates filter expressions and interpretation of variants. Thus, *vembrane* only accepts files whose records have been split such that each alternative allele has its own record. This can, for example, be achieved by normalizing the input with bcftools norm -N -m-any; for consistent results, this is best done before annotation.

To our knowledge, *vembrane* is the first tool to comprehensively handle breakend variants (BNDs): BNDs are a way of encoding structural variants by grouping two or more genomic breakpoints into a joint structural variant *event*. As variant files are usually sorted by chromosomal position, BND records from the same event can occur in distant parts of the file. Thus, even if the event it belongs to is known for each BND at the time of reading it, the total number of BNDs (and all associated annotations) for a specific event remains unknown until reaching the end of the file. *vembrane* thus needs to ensure that each event is removed or kept *as a whole*. While non-BND variants are yielded instantly during iteration, BND processing is deferred until sufficient information is available—this is the case as soon as at least one BND of an event passes the filter expression.

### 2.2 Comparison to other tools

Various tools exist for filtering VCF records using conditional expressions over their fields. They vary greatly in the scope of their functionality (Table 1). For example, the *SnpEff* and *VEP* annotation suites have their own filtering tools, *SnpSift* and *filter_vep*. Both use custom syntax, special handling of only their own annotations and neither supports the BCF format. Additionally, *filter_vep* is several orders of magnitude slower than the other tools (Supplementary Fig. S1). The *bcftools* suite also developed its own expression syntax and supports *VEP* annotations by explicitly activating a dedicated plugin. *bio-vcf* (Garrison *et al.*, 2021) defines its own domain-specific language for processing VCF files, is multi-threaded by default, but has neither BCF support nor built-in support for annotations. *slivar* (Pedersen *et al.*, 2021) is geared more toward trio/pedigree filter scenarios, but has some support for specific parts of *SnpEff*, *VEP* and *bcftools* annotations such as Consequence. The only other tool that does not define its own syntax is *VcfFilterJdk* (Lindenbaum and Redon, 2018), which uses Java expressions for filtering and in principle supports both *VEP* and *SnpEff* (only supports obsolete annotation format from the ‘EFF' tag) annotations. However, at the time of writing, it produced incorrect VCF v4.2 files. A detailed comparison of specific syntactic capabilities of the different tools, as well as a performance benchmark, can be found in the supplementary Material.

**Table 1.** Comparing different tools/properties, see Supplementary Material for details.

Tool	Syntax	Annotation	I/O formats	Breakends	Speed
*bcftools*	Custom	*VEP* ^a^	VCF, BCF	No	+++
*bio-vcf*	Ruby/custom^b^	-	VCF	No	-
*filter_vep*	Custom	*VEP*	VCF	No	---
*slivar*	Js/custom^b^	Custom^c^	VCF, BCF	No	-
*SnpSift*	Custom	*SnpEff*	VCF	No	+
*VcfFilterJdk*	Java	*VEP*, *SnpEff*^d^	VCF, BCF^e^	No	∘^f^
*vembrane*	Python	*VEP*, *SnpEff*	VCF, BCF	Yes	++

a Through +split-vep plugin.

b Additionally ‘*custom*’ because some scenarios require complex Command Line Interface option combinations.

c Special handling of impact annotations from *bcftools*, *VEP* or *SnpEff*.

d EFF only.

e VCF < v4.3, BCF < v2.1 only.

f Manually estimated performance, since it is not included in the benchmark due to incompatible VCF version support and lack of conda packages.

Symbols used for speed classification range from --- (slowest) through ∘ (average) to +++ (fastest).

## 3 Summary

*vembrane* is a software for efficient filtering of data in VCF and BCF files. It combines the capabilities of existing tools and should work as a replacement to any of them. Thus, users will not have to remember which tool can achieve what, but should be able to perform any filtering task with *vembrane*. Further, *vembrane* allows for filtering via arbitrary Python expressions, meaning that Python users can compose filtering expressions without having to learn custom syntax. In addition, it extends beyond existing functionality in other tools by providing support for breakends. Finally, it also allows formatting VCF files into tables and has basic support for annotating records itself.

## Supplementary Material

btac810_Supplementary_Data
